# A Linen Guild's Work at Leeds

**Published:** 1914-05-30

**Authors:** 


### A Linen Guild's Work at Leeds.

The President, Robinia Viscountess Mountgarret, and many Vice-Presidents and friends were present at the sixth annual meeting of the Ladies' Working Guild, which was recently held in the Hospital for Women and Children at Leeds. The Hon. Secretary, Mrs. Massey Richardson, presented her report. The amount of linen received, it appeared from the meeting, was not quite up to the average. The number of articles sent was 342, and the value of the linen with subscriptions amounted to £51 15s. 9d. Since the Guild started, six years ago, the committee have not been called upon to replenish any sheets, draw-sheets, pillow-cases, hand-towels, and tea-cloths, the provision of which saves the hospital a considerable amount each year. Of course, the Guild is still in its infancy, and Lady Mountgarret, on behalf of the Guild, thanked the hon. secretary for her arduous labours, and proposed, by way of widening the interest, the addition of a list of associate members.

